# A comparative study of doctor’s meaning construction in diagnostic discourse with different degrees of patient satisfaction: A review

**DOI:** 10.1097/MD.0000000000042528

**Published:** 2025-05-23

**Authors:** Haiying Liang, Caihong Wang

**Affiliations:** a College of Foreign Languages and Literature, Northwest Normal University, Lanzhou, China.

**Keywords:** doctor–patient interaction, meaning construction, patient satisfaction, Systemic Functional Linguistics, transitivity system

## Abstract

Drawing on the theory of ideational meaning construction in Systemic Functional Linguistics, and using 22,945 words of 4 doctor’s outpatient doctor–patient interaction as its data resource, the paper makes a comparative analysis of doctors’ meaning construction in their diagnostic discourse via transitivity system employed by doctors with higher patient satisfaction and those with lower patient satisfaction. The results show that doctors with higher patient satisfaction choose more behavioral process, attribute and extent, in the process type, participants, and circumstantial elements respectively, indicating they pay more attention to the detailed information of the disease and patients’ daily life in order to give more accurate diagnosis and relieve the pressure of the patients. By contrast, doctors with lower patient satisfaction employ fewer behavioral process, more goal, and more manner, cause and contingency, suggesting they pay less attention to patients’ condition and the degree of patients’ acceptance and participation, instead emphasizing their authority in medicine and the way of treatment. The research findings offer reference to the harmonious relationship establishment between the doctor and the patient.

## 1. Introduction

As institutional discourse, doctor–patient interaction has been a heated topic for researchers. Current research on doctor–patient interaction has been mainly focused on the descriptions of micro characteristics, pragmatic principles, and the influence of social factors on doctor–patient interaction based on theories and methods in Conversational Analysis, Pragmatics, Sociolinguistics, and Systemic Functional Linguistics (SFL).

In the Conversational Analysis, many researchers have put their focus on turn-taking,^[1,2]^ conversational structure,^[3]^ questioning,^[4]^ power asymmetries,^[5,6]^ formulation,^[7,8]^ and empathy.^[9]^ In Sociolinguistics, the major focus is put on gender,^[10–12]^ age, educational background, and social status.^[10,11]^ In Pragmatics, attempts have been made to investigate discourse strategies,^[13,14]^ speech acts,^[15,16]^ communicative intention,^[17]^ politeness principle,^[18]^ cooperative principle,^[19]^ and pragmatic empathy.^[20–22]^

In SFL thereafter, researchers have studied doctor–patient interaction from 3 metafunctions, context, stratification, instantiation, and generic structure. Within metafunctions Mathers^[23]^ and Mcmanus^[24]^ made diachronic studies on ideational meaning construction in information profiles for British patients, Cohen^[25]^ and Mok^[26]^ analyzed interpersonal meaning construction of patients with schizophrenia and dementia. Liang^[27]^ made a comparative study on doctors’ individualized interpersonal meaning construction in terms of gender difference and working experience. Slade et al^[28]^ made a synthetic study of meaning construction in the emergency department. Besides, Matthiessen^[29]^ made a comprehensive analysis on doctor–patient interaction, including context, ideational meaning, instantiation and stratification. Kuang and Li^[30]^ analyzed the generic structure in doctor–patient interaction.

From the current research situation, it can be found that most of the researches mainly analyzed the micro and macro features in doctor–patient interaction by employing theories in Conversational Analysis, Pragmatics, Sociolinguistics and SFL, fewer researches were conducted on the comparison of language features of different doctors’ discourses related to patient satisfaction. Given the current research situation, by employing theories in SFL, the present study attempts to make a comparative analysis of transitivity processes used by doctors with higher patient satisfaction and those with lower patient satisfaction. Based on the different features indicated by doctors’ use of different participants, processes and circumstances, further discussion will be made on the different ideational meaning construction and certain relationship they establish with patients with a view to offering reference to the harmonious doctor–patient relationship construction.

## 2. Research paradigm of ideational meaning in SFL

As appliable linguistics, SFL can be used to solve practical problems in different fields by employing kernel notions in SFL. Regarding language as social semiotics, SFL holds that language is a system of meaning potential, offering various possibilities for people to express meaning in participating in different social activities.^[31]^ According to different purposes people use language in daily life, language can be deployed to construct 3 kinds of meanings: ideational meaning, representing different kinds of experiences in our physical or inner world; interpersonal meaning, establishing certain interpersonal relationship and show our stance and attitude towards people and things; textual meaning, organizing meaning into a coherent discourse by certain devices and expressing meaning in certain context.

Among 3 kinds of meanings, ideational meaning is the meaning as representation. Language enables human beings to build a mental picture of reality, to make sense of what goes on around them and inside them. It is realized mainly by transitivity system in lexcio-grammatical stratum. Halliday and Matthiessen^[32]^ describe transitivity as the system which ‘construes the world of experience into a manageable set of process types. According to Halliday transitivity system, there are 3 main process types realized by the clause: material, mental, and relational. In addition to these 3 main processes, there are further 3 “subsidiary” categories that are recognized in the grammar as being intermediate between 2 of the main process types. They are behavioral processes, verbal processes, and existential processes.

The material process is a process of doing. It expresses the notion that some entity “does” something-which may be done “to” some other entity. The mental process expresses a feeling, thinking, and perceiving. Mental processes characterize processes that occur internally, “in the world of consciousness.”^[32]^ The relational process is a process of being. And there are 3 types: intensive, circumstantial, and possessive. Each of these comes in 2 distinct modes: attributive and identifying. The attributive type is used to ascribe qualities (or possessions), in the identifying variety, it is used to identify 1 entity in terms of another. Behavioral Process is the process of (typically human) physiological and psychological behavior, like breathing, coughing, smiling, dreaming, and staring. Verbal Process is concerned with “saying” and ‘conveying messages’. Existential Process represents that something exists or happens. Unlike the relational or material processes, the participant in an existential process is not represented as being involved in any kind of “going on.”^[33]^

When people choose certain process types to represent experiences, they are restrained and influenced by cultural and situational context, including social activity they are attending in cultural context and the major contents in a certain activity in situational context. By analyzing the features of process types, participants and circumstantial elements in transitivity system employed by different kinds of doctors, we can detect what kinds of experiences they prefer to represent, what types of participants they choose to classify, and what kinds of circumstances they prefer to use, based on the distributive features, thereafter detecting what kinds of relationship they keep with patients.

## 3. Research design

In order to get authentic data, the researchers went to 1 hospital in a certain province in China and invited 8 doctors and their patients as participants under the condition of their consent. To avoid other variables to influence the research results, the factors of gender and working experience are restrained, and only male and experienced doctors were chosen as our participants. In this process, patients came to the outpatient department to ask for help, and doctors interacted with patients to give diagnoses and prescriptions. After the consultation, the patients were invited to complete the questionnaire to see whether they were satisfied with their doctors, which were filled in out of the clinic and independently by the patients. The questionnaire we adopted is Patient Satisfaction Questionnaire (PSQ),^[34–36]^ which is the most widely used questionnaire in the study of patient satisfaction. The PSQ developed by Ware et al^[34]^ over a decade for the National Center for Health Services Research provided the foundation for PSQ-III.^[37]^ The PSQ-III consists of 50 items, which are divided into 7 aspects, including General Satisfaction, Technical Quality, Interpersonal Aspects, Communication, Financial Aspects, Time Spent With Doctor, and Access/Availability/Convenience. Among 50 items, 12 items related to Communication and Interpersonal Aspects are chosen in terms of research purpose. The correlations, validity and reliability of the questionnaire are tested, and reliability estimates range from 0.77 to 0.89 in the MOS baseline sample.

The entire recording process was anonymous and private. Therefore, the conversation between doctors and patients was relatively natural without other’s interruption and influence. The recording time of 1 doctor is about 6 to 8 hours, so the total recording time is approximately 48 to 64 hours. After collecting the questionnaires filled in by the patients, the authors removed the invalid questionnaires and got 149 questionnaires finally, by which we rated the degree of patient satisfaction of each doctor respectively according to the results of questions. Finally, the top 2 doctors with the highest patient satisfaction and 2 with the lowest patient satisfaction were chosen as the subjects of our study (see Table 1).

**Table 1:** Background information of 4 doctors and corpus capacity transcribed.

Doctor	Gender	Age	Department	Title	Total number of words with patients	Number of doctor’s words
Doctor 1 (DL1)*	Male	57	Orthopedics	Chief Physician	5996	3988
Doctor 2 (DH1)	Male	59	Spleen and stomach department	Chief Physician	5770	1361
Doctor 3 (DL2)	Male	57	Spine orthopedics	Chief Physician	5694	3538
Doctor 4 (DH2)	Male	54	Spleen and stomach department	Associate Chief Physician	5485	1918

This table describes respectively the kernel information of 4 participants in this study and lists the concrete total number of words in the communication with patients and number of doctor’s words.

DL = doctor with lower patient satisfaction; DH = doctor with higher patient satisfaction.

* Doctor 1: In order to protect the privacy of the doctor, the author employs it as reference. DL1: In the following examples, in order to show the source of the conversation, the author employs DL1 to stand for doctor 1 with lower patient satisfaction and DH1 for doctor 1 with higher patient satisfaction.

Then came the process of transcription of the doctor–patient interaction of these 4 doctors. The transcription standard is the oral transcription standard of Eggins and Slade^[38]^ and Hasan.^[39]^ The authors adopted a broad transcription, focusing on the language use itself, which is different from the narrow transcription method of the Conversational Analysis. After the transcription was finished, the authors got the doctor–patient conversation with 22,945 words. In doctor’s discourse with higher patient satisfaction, 11,255 words are calculated and the number of doctors’ words is 3279, occupying 29% of the total number of doctor–patient interaction, while among 11,690 total words in doctor’s discourse with lower patient satisfaction doctors’ words are 7536, occupying 64%. It indicates that the doctors with higher patient satisfaction offer more room for patients’ contribution to the interaction, as Roter and Hall^[11]^ finds, greater physician dominance predicts lower satisfaction. As for the doctors with high patient satisfaction, they give the patient more time and space to talk about his disease, lifestyle, and other daily issues related to disease, whereas as to those with lower patient satisfaction, they mainly ask relevant questions and offer treatment plans.

After transcription, the researchers mainly employed UAM Corpus Tool and SPSS to deal with data transcribed. First, the statistics of the transitivity process, participants and circumstantial elements were exported from the UAM. Then detailed comparison of 2 kinds of data in doctor’s discourse was made. Then the Chi-square test in SPSS was used to know if there is a significant difference in doctor’s discourse with higher patient satisfaction and those with lower patient satisfaction.

## 4. Transitivity analysis employed by doctor with higher patient satisfaction (DH) and doctor with lower patient satisfaction (DL)

After labeling the processes, participants, and circumstantial elements, the detailed data are exported and listed in Table 2.

**Table 2:** Transitivity analysis of doctor’s discourse with different degrees of patient satisfaction.

Transitivity system		Doctors’ discourse with higher patient satisfaction	Doctors’ discourse with lower patient satisfaction		Doctors’ discourse with higher patient satisfaction	Doctors’ discourse with lower patient satisfaction
Processes	Material	169	432	Existential	11	30
	Relational	92	196	Behavioral	7	2
	Mental	30	66	Verbal	2	13
	Attribute	174	187	Recipient	3	37
	Goal	121	244	Verbiage	2	6
	Carrier	99	151	Sayer	1	10
	Phenomenon	28	22	Target	1	1
Participants	Senser	25	41	Receiver	0	9
	Identifier	17	34	Behaver	0	2
	Identified	16	32	Scope	0	0
	Actor	15	116	Behavior	0	0
	Existent	10	30	Client	0	0
	Beneficiary	3	46			
	Location	58	156	Manner	3	50
Circumstantial elements	Extent	43	75	Cause	1	20
	Accompaniment	10	17	Role	0	1
	Contingency	6	46	Matter	0	0
	Angel	4	16			

This table mainly describes the concrete frequency of 2 kinds of doctors’ employment of transitivity system, including the employment of process types, participants, and circumstantial elements.

Table 2 mainly reveals the concrete features of processes, participants, and circumstantial elements in doctors’ discourse with different degrees of patient satisfaction. To start with, among 6 process types, doctors with higher patient satisfaction employ material process for the most, then followed by relational, mental, existential, behavioral, and verbal processes. There are similarities and differences in the choices of process types for doctors with lower patient satisfaction. The similarities lie in the same choices of the first fourth processes, which complies with the characteristics of doctor–patient interaction. The above-mentioned processes are mainly adopted to ask patients to do as they say, and know the condition of patients’ situation and the disease itself, which indicates that doctors employ adequate professional knowledge to make diagnoses and treatments^[40]^ The difference lies in that the fifth process used by DH is the behavioral process while it is the verbal process by DL. And the last process by DH is the verbal process while it is the behavioral process by DL. It may result from that DH pay much attention to patients’ description of the disease, the patient’s daily life and feeling, which is good for the doctor to collect more useful information in the diagnosis. By contrast, by employing verbal process, DL put much focus on the contents of their words, and ask patients to listen to their treatment.

Then, in doctor’s discourse with higher patient satisfaction, the most frequently chosen participants include Attribute, followed by Goal, Carrier, and Phenomenon, while Goal, Attribute, Carrier and Actor in those with lower patient satisfaction are deployed. These findings are correspondent with the use of process types. Finally, the most frequently used circumstantial elements are Location and Extent in doctor’s discourse with higher patient satisfaction and those with lower patient satisfaction. They reveal the features of doctor–patient interaction. In doctor–patient interaction, the doctor asks more about the duration of diseases and the painful location.

In the following sections, the detailed employment of processes, participants and circumstantial elements are analyzed.

### 4.1. Transitivity processes employed by DH and DL

**Figure 1.**
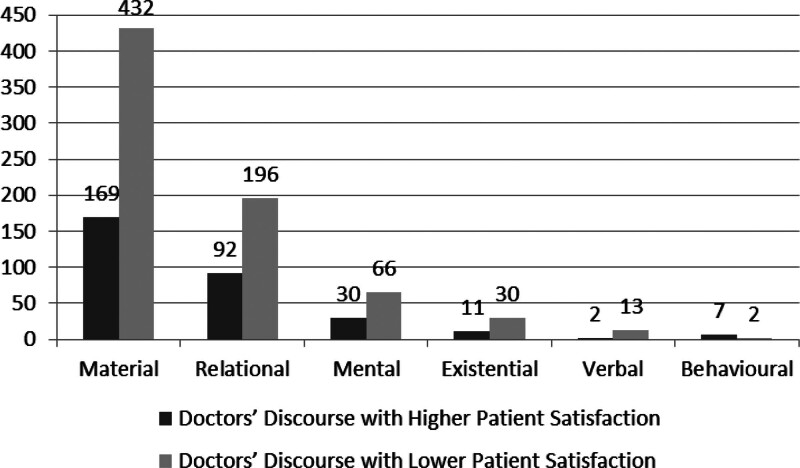
Six processes distributed in doctor’s discourse with different degrees of patient satisfaction. This diagram shows the different frequency of 6 process types used by doctors with higher patient satisfaction and those with lower patient satisfaction.

Figure 1 demonstrates the transitivity process employed by doctors with different degrees of patient satisfaction.

(In Chi-square test, *P* = .018, *P* < .05, indicating there is a significant difference in the choice of transitivity process with different kinds of doctor’s discourse.)

As shown in Figure 1, the material process is deployed the most, followed by relational process, mental process and existential process in both types of doctors’ discourse. Regarding the verbal process, it is the least in doctor’s discourse with higher patient satisfaction, while in doctor’s discourse with lower patient satisfaction, the behavioral process is the least. Based on Chi-square test, we calculated the adjusted residue (AR thereafter) result by SPSS to find out the specific difference within transitivity system, the result of which shows that there are no significant significance in the employment of 5 processes, lower than 1.96 (AR<|1.96|) (According to the explanation of Adjusted Residue, if the absolute value of Adjusted Residue is higher than 1.96, significant difference exists between 2 groups of data.), with material process - (“-”: To show that DL employs more resources than DH.)1.2, relational 1.0, mental 0.4, existential -0.4, and verbal -1.4, while the result of behavioral process is -3.2, indicating that the big difference mainly lies in the employment of behavioral process, doctors with lower patient satisfaction choose fewer behavior process.

Specifically speaking, in the doctor’s discourse with higher patient satisfaction, the material process is employed the most, and the next is the relational process, mental process, and the existential process. Material process and relational process are more objective and persuading to express objective knowledge and real experience,^[41]^ which are beneficial for patients to trust doctor’s authority. Thompson^[33]^ indicates that the material process involves physical actions, which mainly refer to do things. By using the material process, the doctor employs “eat, take, give, and teach” to give the diagnosis and treatment. The relational process refers to a relationship set up between 2 concepts. Although the relational process lacks coordination among people, it could show the characteristics of concrete concepts and clear expression.^[42]^ The doctor employs “is, has” to state the attribute and characteristics related to the disease with a view to giving more accurate diagnoses. For example:

(1)D: Ni zhè shì wèi jìngluán, bùnéng chī lěng de, jiǔ bùnéng hē.

You this is gastro spasm can’t eat cold alcohol can’t drink

Chī diǎn zhōngyào tiáo yīxià ba, là de yě bùnéng chī.

Eat some Chinese medicine to regulate spicily yet can’t eat

“You had gastro spasm, so you couldn’t eat cold food and drink alcohol. You could make some medicine to regulate. Besides, you couldn’t eat spicy food.”

P: Làjiāo yě bùnéng chī?

Pepper yet can’t eat

“I couldn’t eat pepper?” (DH2)

In Example (1) DH2 employs material process and relational process. First, the doctor uses words like “eat, drink and make” to tell the patient not to eat spicy food and drink alcohol. Moreover, the doctor gives a prescription and asks the patient to take some medicine. It shows that doctor gives the patient suggestions and prescriptions, which states her responsibility and concern. Second, “have” is used to give a diagnosis to the patient after knowing more about the patient’s situation. In this example, the relational process employed by the doctor indicates the doctor has professional medical knowledge and shares it with the patient. In a word, giving more suggestions and information to the patient and providing technical and professional medical knowledge could shorten the distance between doctor and patient, and invite the patient to participant in the whole process.

(2)P: Wǒ shì juédé yī dào 150, xuěyā jiù gāole.

I is think arriving 150 blood pressure is high.

“I think that blood pressure is 150, it’s high.”

D: Bù gāo, gāoyā gāo diǎn bù pà, dīyā

No high High pressure high a little not afraid low pressure

dīle jiù wéixiǎn ne, gāoyā dào 150 méiyǒu wéixiǎn.

low is dangerous high pressure arrive 150 without danger

“It’s not high. You don’t have to be afraid of higher blood pressure. But lower blood pressure is more dangerous, while high blood pressure more than 150 is not dangerous.” (DH2)

In Example (2), the main process used by DH2 is the relational process. The doctor employs “Lower pressure is dangerous,” “The high blood pressure more than 150 is not dangerous” to tell the patient more about her situation concretely. The doctor mainly tells the patient about her blood pressure patiently. Sometimes, patients may feel nervous when they are sick, and the doctor’s relieving words could make them feel better and at ease. At the same time, the patient could know more about her disease and the current situation so that she could pay more attention to her situation and follow the doctor’s advice.

The mental process is the third process, which is a process of sensing. The function of mental process is mainly to pay more attention to the patient’s emotional aspects. The doctor–patient relationship is an intrinsically high-context phenomenon, within which the communication of expert knowledge and emotions are central.^[11]^ Roter and Hall^[11]^ also indicates that the doctor should put the patient at ease and express some degree of warmth and friendliness. In the diagnostic process, the doctor usually asks patients about their pain location and pain degree, and to some degree, he/she knows about patients’ pain and their feeling. Moreover, the doctor also gives a diagnosis and treatment to help the patient relieve the pain. All these show the doctor is concerned and serious about the patient. For example:

(3)D: Màn man de, néng dàbiànle zàishuō. Zuǐ lǐ kǔ bù?

Slowly can defecate again say Mouth inside bitter not

“Slowly, you can take a shit then we deal with other symptoms. Do you feel bitter in your mouth?”

P: Kǔ ne, zuìjìn kǔ de lìhài.

Bitter recently bitter seriously

“Yes, recently I feel bitter seriously in the mouth.” (DH1)

(4)D: Shǒujiǎo lěng bù lěng?

Hands feet cold not cold

“Are your hands and feet cold?”

P: Bù.

No

“No.” (DH1)

In Examples (3) and (4), DH1 uses the mental process. “Bitter and cold” are employed to know more about the patient’s subjective feelings. At the same time, the doctor cares more about the patient’s feelings, which would make the patient relaxed and want to tell the doctor more about his mental situation, his emotional change, and personal feeling. Moreover, emotional communication is as important as language communication. By knowing more about the patient’s feelings, thought and desires, the doctor can get overall information, so the diagnosis and treatment will be effective and trustable.

The fourth process is the existential process. The existential process is mainly used to state fact objectively and express something existed. It expresses the mere existence of an entity without predicting anything else of it.^[33]^ Patients who get more information are more satisfied than patients who get less.^[43–45]^ The existential process is mainly used to state or describe something objectively in the real world without concerning with any emotional expression and actions. And “be” is usually employed to state the objective existent. By using existential process, the doctor attempts to give or get objective description of the patients’ disease.

(5)D: Shí jǐ tiān yě méiyǒu dàbiàn ma? Píngshí wèi yǒu wèntí

Ten days yet not has feces Usually stomach has problems méi, yǒu méiyǒu wèibìng.

not have not have stomach illness

“You have no feces for more than ten days. Do you have problems in the stomach or do you have illness in the stomach?” (DH1)

For Example (5), “have no feces, have problems and have an illness” are used by the doctor to know about the patient’s symptoms and medical history. The whole process is objective and real in the diagnostic process without any personal feeling, which is beneficial for the patient to trust the doctor’s professional knowledge.

The fifth process is the behavioral process, the process of (typically human) physiological and psychological behavior. By employing the behavioral process, the doctor tries to ask the patient about their sleeping, diet and breathing to obtain more information about the disease. At the same time, the patient also feels that doctor cares more about their daily life and talks more about their situation, which will create a relaxing and harmonious atmosphere for both of them.

(6)D: Tiānqì lěng ké dé lìhài háishì rè ké dé lìhài?

Weather cold cough seriously or hot cough seriously

“You cough seriously on a cold day or hot day?”

P: Tiānqì lěng de shíhòu, yóuqí shì yīn tiān.

Weather cold time especially is cloudy day

“When it is cold, I cough seriously, especially on the cloudy day.” (DH1)

(7)D: Wǎnshàng néng shuìzhe ma?

Night can sleep

“Can you sleep at night?”

P: Shuìmián bù tài hǎo.

Sleep not well

“I can’t sleep well.” (DH1)

In Examples (6) and (7), the doctor mainly employs the behavioral process to know more about the patient’s current situation, her daily life and other related aspects. By employing words like “cough, diet, sleeping, menstruation, and urination,” the doctor on the 1 hand gets more information provided by the patient. On the other hand, this kind of conversation could make a close relationship between doctor and patient.

The sixth process is the verbal process. It is a process of saying, and it covers any kind of symbolic exchange of meaning.^[31]^ Physicians have to share their medical expertise with patients in such a way that this information is clear, relevant, and useful to patients.^[11]^ Because the doctor is professional and responsible, their suggestions, prescription and treatment plan are essential for patients.

(8)D: Zànshí bù gǎn biàn, děng hòulái zhèngchángle wǒ gàosù

Temporarily no dare change wait later normal I tell

nǐ jiǎn shénme nǐ zài jiǎn.

you reduce what you again reduce

“Temporarily no change, I will tell you what you should not eat after it is normal.”

P；Ń ń, zhì yǎn de yào háishì chī shàng ba.

Um, treat eyes medicines also eat

“Um, you also eat medicine to treat your eyes.” (DH2)

In Example (8), the doctor uses “I tell you” to show that the medical transcription should be based on the doctor’s advice. It could indicate the doctor’s responsibility and seriousness in her profession.

Similar to the employment of process types in the discourses with higher patient satisfaction, doctors with lower patient satisfaction choose 5 similar process types including material, mental, relational, existential and verbal process. The DL choose these similar processes to give suggestions and treatment, and try to understand patients’ situation, which conform to the characteristics of institutional discourse.

The differences of the employment of process types deployed by 2 types of doctors lie in the behavioral process used by DL. According to the AR result (-3.2) indicating the significant difference between 2 types of doctors, DL doctors choose much fewer behavioral process. The behavioral process mainly stresses that doctor is concerned more about patient’s behavior and daily life (As shown in the previous part), but the doctor uses the least behavioral process among 6 processes. Most often, patients are asked about their satisfaction with the humaneness or interpersonal qualities of their doctors, and least often about their satisfaction with the attention given to personal (not medical) problems or problems of living.^[46]^ By adopting fewer behavioral process, the doctor may not get holistic information of the patient. Therefore, doctors should try to use more behavioral processes to make patients satisfied. By asking and caring about patients’ daily life, a harmonious doctor–patient conversation can be created and the distance between them can be closer.^[27]^

### 4.2. Participants employed by DH and DL

**Figure 2.**
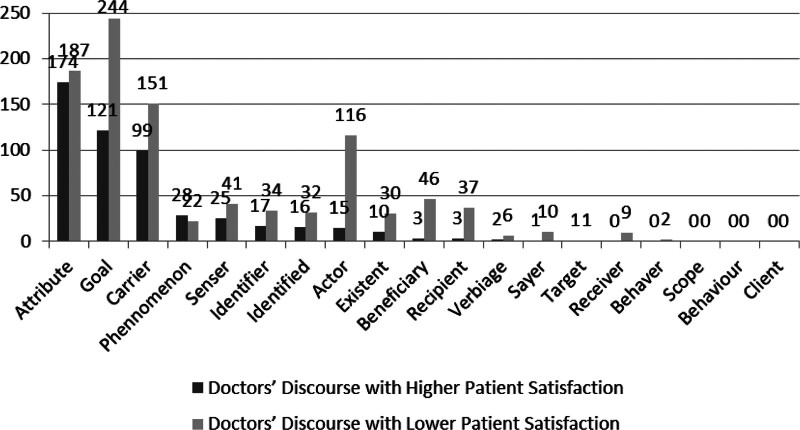
Participants distributed in doctor’s discourse with different degrees of patient satisfaction. This diagram shows the different frequency of 20 types of participants used by doctors with higher patient satisfaction and those with lower patient satisfaction.

Figure 2 displays the frequency of participants deployed by DH and DL.

(In Chi-square test, *P* = .000, *P* < .05, it states that there is a significant difference in transitivity process in different kinds of doctor’s discourse.)

Combined with AR results, the major differences of participants (AR>|1.96|) used by 2 types of doctors are ranked as Attribute (6.2), Actor (-5.9), Beneficiary (-4.3), Recipient (-3.7), Phenomenon (3.2), Receiver (-2.2). That is to say, DH choose more Attribute and Phenomenon while DL adopt more Actor, Beneficiary, Recipient and Receiver.

In DH’s discourse, The Attribute (174) is the most frequently used, which belong to relational process. It is important to appreciate the tremendous power of permitting a patient to orient his or her physician to the patient’s “lifeworld”: to tell the patient’s stories and share the patient’s experiences, understanding, theories, and concerns about health.^[47]^ By attributive relational process and the Attribute, the doctor may have a better understanding of what would be of the patients’ daily life.

(9) D: Yāo kùn bù?

Waist tired not

“Does your waist feel tired?”

P: Lái yuèjīng qián jǐ tiān yāo huì suān.

Coming menstruation before several days waist can sore

“I feel sore in the waist before menstruation.”

D: Duōshǎo suìle?

What age

“How old are you?” (DH2)

In Example (9), “tired and how old” are used by the doctor to know some basic information about the patient and his disease. The Attribute is employed more by doctors to describe and know the real and objective situation, which shows the doctor has rich medical knowledge and experience. At the same time, the patient would trust the doctor, so he could tell more about his disease and needs, which will be helpful for the doctor to give a right and overall diagnosis and proper treatment.

Besides, DH also choose more Phenomenon in their discourse. Phenomenon is a participant in mental process. By adopting more Phenomenon, the doctor gives a more detailed explanation or diagnosis of patient’s disease or puts forward his own suggestions.

(10): Wǒ juédé zhège fāngàn shì hélǐde, érqiě tǐng yǒuxiàode.

I think this plan is reasonable, and effective

“I think this plan is reasonable and effective.” (DH2)

(11): Wǒ huì zài pínggū yīxià. Wǒ jiù hàipà chūxiàn línbā zhuǎnyí.

I will again assess. I was afraid of lymphatic metastasis.

“I will assess it again. I was afraid of lymphatic metastasis.” (DH1)

In Example (10), the doctor chooses Mental process to give the patient his own suggestion, and Phenomenon is “this plan is reasonable and effective”. By using verb “think”, the doctor emphasizes his suggestion is one of the possible solutions, expanding dialogic space between the doctor and patient.^[48]^ In Example (11) the verb being “be afraid of” and combining with Phenomenon “lymphatic metastasis”, the doctor gives his judgment and mitigates his tone of voice by considering the degree of patient’s acceptance.

By contrast, DL employ more Actor, Beneficiary, Recipient and Receiver. The first 3 participants belong to material process, which mainly functions as asking patients to do what they are told, which, to a certain degree, lacks the degree of acceptance and doesn’t account for the willingness of the patients.

(12) P: Fāngbiàn zhene.

Convenient

“It is convenient.”

D: Nà wǒ gěi nǐ kāi xiē zhōngyào. Qiánmiàn wǒ gěi

That I give you take some Chinese medicine Before I give dàjiā jiào jǐngchuí cāo, nǐ xuéle ma?

others teach cervical exercises you learn

“Then I take some Chinese medicine to you. I teach others cervical exercises before. Do you learn it?” (DL1)

In Example (12), the doctor employs Goals like “traditional Chinese medicine and cervical exercises” to give treatment and make the prescription. “You” is Recipient indicating that the patient is the receiver and has to accept the doctor’s treatment, and “others” as Beneficiary indicating others may benefit from the doctors’ suggestions.

Receiver is a participant in verbal process. By adopting verbal process and Receiver, the doctor tries to emphasize his own treatment and authority in medicine.

(13): Wǒ gěinǐ jiǎng, méiyǒu biéde bànfǎ, jiùshì zházhēn shì wéiyī de bànfǎ. Zuò

I you tell, no other way, needle is only way.

hécí méiyòng.

NMR useless.

“Let me tell you that there is no other way, but a needle is the only way. NMR is useless” (DL2)

In Example (13), the doctor tells Receiver “you” the only choice he has, which emphasizes the authority of the doctor and contracts the dialogistic space between the doctor and patient, for there is little negotiation of the doctor’s ways of treatment.

### 4.3. Circumstantial elements employed by DH and DL

**Figure 3.**
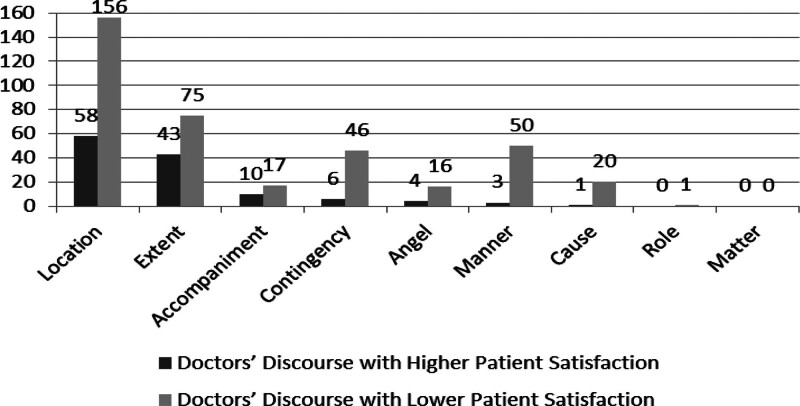
Circumstantial elements distributed in doctor’s discourse with different degrees of patient satisfaction. This diagram shows the different frequency of 9 circumstantial elements types used by doctors with higher patient satisfaction and those with lower patient satisfaction.

Figure 3 displays the frequency of circumstantial elements deployed by DH and DL.

(In Chi-square test, *P* = .000, *P* < .05, it states that there is a significant difference in transitivity process in different kinds of doctor’s discourse.)

Combined with AR results, the significant differences (AR>|1.96|) mainly lie in the use of Extent (3.4), Manner (-3.4), Contingency (-2.3), Cause (-2.2), showing that DH employ more Extent, while DL choose more Manner, Contingency and Cause.

Generally, the DH use more temporal adverbial and adverbial adjuncts like Extent to ask about the patient’s disease and explain his treatment in a more concrete way in order to make the patients rest assured.

(14) D: Yòng jīguāng, dàgài 5000 kuài qián, sìwǔ tiān

Use laser about 5000 Yuan four five days

jiù kěyǐle. Guāng chī yào wǒ zhè’er méiyǒu, chī bù hǎo,

Ok Only eat medicine I there not have eat not well

nǐ kàn kàn biérén nà’er yǒu méiyǒu, wǒ zhè’er méiyǒu.

you see others there have not have I there not have

Nǐ xiànzài niánqīng yǒu jīhuì, děngguòle zhège shījiān

You now young have opportunity wait after this period

jiù zuòbùliǎole. Yǒuxiē rén gāng kāishǐ xiǎng bùtōng, guòle

does not someone just now start think not smooth after

sān gè yuè yòu yào zuò, dànshì yǐjīng zuòbùliǎole, yīn wéi

three months again do but already do not because

yǐjīngguòle zhège shíjiānle.

“Using laser takes about 5,000 RMB. And four or five days can complete it. I have no medicine only to treat, and it doesn’t work out. You have the opportunity to recover because you are young. After this period, you cannot do it. Someone at the beginning doesn’t want to do it, but three months later they want to do it without opportunity because time has passed.” (DH1)

In Example (14), the DH uses duration in Extent like “four or 5 days,” “this period” and “three months” to give his advice according to the patient’s current situation. Because the symptoms change as time goes by, the doctor adopts the best ways in the treatment process. This shows the doctor has rich medical knowledge and experience, and offer concrete and proper treatment.

By contrast, DL employ more Manner. The circumstantial element of Manner comprises 3 subcategories: Means, Quality, Comparison.^[31]^ By using relevant preposition phrases and adverbial phrases of Manner, the doctor emphasizes the way of his treatment, stressing his authority and status to a certain degree.

(15) D: Bù zhǔn huàn xié. Jiù zhège xié de gāodù, nǐ shìdàng de zǒu,

No allow change shoe this shoe height you proper walk

bùyào qiángxíng de zǒu.

no force walk.

“No change of shoes. Just keep the height of this shoe, and you should walk appropriately, instead of walking forcibly.”

P: Zěnme chī ya?

How eat?

“How to eat this medicine?” (DL1)

In this example, the doctor uses words like “appropriately” and “forcibly” to show the manner of exercise. The words said by the doctor are in a commanding tone, which will give the patient a sense of oppression.

Besides, DL employ more Cause and Contingency to explain the reason and the contingent situation of the disease.

(16) wúlùn yuányīn shi shá dōuba yào chīshàng. Nǐ míngtiān

No matter reason is what both eat medicine You tomorrow

chī wán yào guòlái wǒ zài kàn kàn, míngtiān

eat finish medicine come I again see Tomorrow

chī shàng yào guòlái liàng yīxià xiěyā zàishuō, jīntiān zhǐ

eat medicine come measure blood pressure Today only

néng zhā yāo hé tuǐ, xuěyā tài gāo, bùnéng zhā bózi.

can needle waist and legs blood pressure too high can’t needle neck

Xiān jìnqù ba.

First enter.

“You have to eat medicine no matter what reason it is. Tomorrow I will give you a diagnosis after you take medicine. And I also check your blood pressure. Today I can only needle your waist and legs because your blood pressure is too high, and the neck cannot be needled. Now you can enter that room.” (DL2)

In Example (16), the doctor uses Contingency “no matter what reason it is” to show his own authority and forcibility which lacks the consideration of patient’s acceptance. And Cause “because your blood pressure is too high” is employed to explain why his treatment is the only option.

## 5. Concluding discussion

From the above analysis, there are similarities and differences in doctor’s discourse with different degrees of patient satisfaction. In the transitivity process, the similarities lie in that doctors employ the material process, relational process, mental process, existential process and verbal process from the most to the least both in doctor’s discourse with higher patients’ satisfaction and those with lower patients’ satisfaction. It reveals the characteristics of doctor–patient interaction, indicating that the major purpose for the doctor is express his opinion objectively and tells the patient what he should do. The main difference consists in doctors with lower patient satisfaction employ fewer behavioral processes, which means that doctors care less about patients’ daily activities and behaviors.

As to the participants, the differences mainly lie in that the Attribute is the most frequently chosen in doctor’s discourse with higher patient satisfaction, which shows that doctors care more about patients’ situations and give an objective diagnosis according to patients’ symptoms. However, the Goal is the mostly used in doctor’s discourse with lower patient satisfaction, indicating that the doctor is in an authoritative position with his medical knowledge, and tries to give orders directly to the patients in the medical aspects.

In circumstantial elements aspects, doctors with higher patient satisfaction choose more Extent to get more information about the patient’s disease and explain his treatment in a more accurate way, while doctors with lower patient satisfaction employ more Manner, Cause and Contingency, paying much attention to their own opinions with less consideration of patients’ participation and acceptance.

By comparing the transitivity processes, the behavioral process is used more often in doctor’s discourse with higher patient satisfaction compared with doctor’s discourse with lower patient satisfaction, so the doctor could try to use more words to express patients’ behavior and their life rather than only ask necessary medical questions. At the same time, they can give patients more time and space to tell them more information. In participants, the Attribute is used more frequently in doctor’s discourse with higher patient satisfaction, while the Goal is used more often in the doctor’s discourse with lower patient satisfaction. Therefore, the doctor could use more objective words to show their specialty and professional skills. In circumstantial elements, Extent is used more frequently by doctors with higher patient satisfaction while Manner, Cause and Contingency are more frequently used by doctors with lower patient satisfaction. The happening of the disease is a long time in different locations of people’s body, so asking related and detailed questions is the essential part for doctors and they can add more information to make the patient feel ease and comfortable.

What is worth mentioning is that, as in the above analysis, there are some common features shared by all doctors, just as the findings in the research on the effect of doctors’ verbal suggestion on patients’ perception of post-needling process^[49]^ and on the subjective perception of effects of the radiofrequency parameters treatment by both patients and doctors.^[50]^ These research findings show that there are no differences between groups on the intensity of post-needling soreness or tenderness over a 1-week follow-up among groups with doctors’ positive, neutral, and negative verbal suggestions, and no changes in the satisfaction of both the patients and the professional, which may be due to patients’ chronic diseases. That indicates that in a certain situation doctors’ verbal expression may not influence the feeling and experience on patients, nevertheless, in more common situations, doctors’ linguistic expressions may exert effects on patients’ understanding of the disease, and their cooperation, and even the curing process of the disease, thus doctors should choose linguistic resources which are more beneficial to establish more equal and harmonious relationship with patients.

## 6. Clinical implications

The study adopts quantitative and qualitative analysis to investigate the employment of linguistic resources in transitivity system by doctors with different degrees of patient satisfaction. From the analysis, it can be revealed that in a clinical situation and in diagnostic procedure, some suggestions could be employed by the doctor to promote a harmonious doctor–patient relationship in terms of doctor’s discourse with higher patient satisfaction. At the same time, some aspects could be avoided by the doctor in the diagnostic procedure in terms of doctor’s discourse with lower patient satisfaction.

Some strategies could be employed by doctors to build an amiable relationship in a common sense. First, the doctor should give space for the patient to tell their behavior and daily life so that patients feel they are respected and concerned in this procedure. The conversation is not only about the disease, but also other topics like emotions, diet, sleeping, and so on. In this way, empathy and rapport can be deeper on the doctor’s side. Second, the doctor can explain medical concepts clearly by moving between technical (medical) and common-sense (everyday) language and provide explicit explanations to patients about processes and procedures in the whole process, so they can use medical expertise in the diagnostic process. Meanwhile, the patient will trust them more and get better professional treatment. Third, the doctor can try to be more patient and friendly to the patient. At the same time, responsibility and giving positive feedback are necessary. In contrast, the doctor shouldn’t say too much, giving the patient more time to describe their disease, behaviors and daily life, so it is beneficial for the doctor to know more information and give a proper diagnosis. Besides, he shouldn’t talk in a strong tone, but in a softer tone and invite the patient to participate in the conversation.

When doctors incorporate these strategies into their medical expertise and practice, patients’ subjective experiences are affected in a much more positive way. And the doctor–patient relationship can be better and harmonious.

## Acknowledgments

Great thanks are given to the doctors and patients willing to participate in the study, and the editors, peer reviewer for this article.

## Author contributions

**Conceptualization:** Haiying Liang.

**Data curation:** Caihong Wang.

**Writing – original draft:** Haiying Liang, Caihong Wang.

**Writing – review & editing:** Haiying Liang.

## References

[1] Maynard D. On clinicians co-implicating recipients’ perspective in the delivery of diagnostic news. In: Drew P. Heritage J., eds. Talk at Work: Interaction in Institutional Settings. Cambridge: New York, USA; 1992:331–358.

[2] Heritage J. The interaction order and clinical practice: some observations on dysfunctions and action steps. Patient Educ Couns. 2011;84:338–43. 21715125 10.1016/j.pec.2011.05.022

[3] Niu L. Study on the structure of doctor-patient conversation in outpatient departments. [dissertation thesis]. Wuhan, China: Central China Normal University; 2014.

[4] Heritage J Robinson J. The structure of patients’ presenting concerns: physicians’ opening questions. Health Commun. 2006;19:89–102. 16548700 10.1207/s15327027hc1902_1

[5] Liu F. Analysis of symmetry and asymmetry in physician-patient conversation. [master’s thesis]. Guangzhou, China: University of International Business and Economics; 2003.

[6] Zheng H. Asymmetry: Makes or breaks a conversation analysis of doctor-patient encounters in Chinese medical settings. [master’s thesis]. Guangdong: Guangdong University of Foreign Studies; 2005.

[7] Gafaranga J Britten N. Formulation in general practice consultations. Text. 2004;24:147–70.

[8] Yu GD. A conversational analysis study of formulation in doctor–patient communication. Foreign Lang Educ. 2009;3:13–9.

[9] Wynn R Wynn M. Empathy as an interactionally achieved phenomenon in psychotherapy characteristics of some conversational resources. J Pragmat. 2006;38:1385–97.

[10] Waitzkin H. Information giving in medical care. J Health Soc Behav. 1985;26:81–101. 4031436

[11] Roter D Hall J. Doctors Talking with Patients/Patients Talking with Doctors: Improving Communication in Medical Visits. Westport, USA: Auburn House; 2006.

[12] Menz F Lalouschek J Reisigl M.. The Representation of Pain and Illness Narratives: Questions of Orality, Gender, and Transformation. Oxford, UK: Blackwell; 2006.

[13] Adegbite W. The structure of texts from herbalist-client encounters in Yoruba traditional medicine. Text. 1995;15:271–97.

[14] Valero-Garces C. Interaction and conversational constrictions in the relationships between suppliers of services and immigrant users. Pragmatics. 2002;12:469–95.

[15] Adegbite W Odebunmi A. Discourse tact in doctor–patient interactions in English: an analysis of diagnosis in medical communication in Nigeria. Nordic J Afr Stud. 2006;15:499–519.

[16] Li HZ. A study on the power inequality of doctor-patient conversation from the perspective of Speech Act. [master’s thesis]. Dalian, China: Dalian University of Technology; 2011.

[17] Li M. A study of outpatient doctor–patient conversation based on communicative intention. [dissertation thesis]. Changchun, China: Jilin University; 2018.

[18] Wang JJ. Questions and power relationships in doctor-patient conversation. J PLA Univ Foreign Lang. 2002;5:10–4.

[19] Jiang J. The use of maxims for cooperation in Chinese medical interviews. Health Commun. 1999;11:215–22.

[20] Bylund C. Empathic communication in the physician-patient encounter. Unpublished Ph.D. Dissertation at Northwestern University: Xian, China; 2001.

[21] Eide H Frankel R Haaversen A. Listening for feeling: identifying and coding empathic and potential empathic opportunities in medical dialogues. Patient Educ Couns. 2004;54:291–7. 15324980 10.1016/j.pec.2003.09.006

[22] Wei CY. A study of pragmatic empathy in doctor-patient communication. J North Univ China (Soc Sci Ed). 2015;3:73–8.

[23] Mathers M. Some evidence for distinctive language use by children with attention deficit hyperactivity disorder. Clin Linguist Phon. 2005;19:215–25. 15823957 10.1080/02699200410001698643

[24] Mcmanus J. The ideology of patient information leaflets: a diachronic study. Discourse Commun. 2009;3:27–56.

[25] Cohen I. The expression of schizophrenia through interpersonal systems at the level of discourse semantics. Unpublished Ph. D. Dissertation at Bar-Ilan University, Israel; 2011.

[26] Mok ZWY. The linguistic construction of interpersonal processes among people with dementia: an application of systemic functional linguistics. Unpublished Ph.D. Dissertation at the University of Louisiana at Lafayette, USA; 2011.

[27] Liang HY. A Study on the Individualized Meaning Construction of Doctor’s Diagnosis and Treatment Discourse in Doctor-Patient Conversation. Beijing: China Social Sciences Press; 2019.

[28] Slade D Scheeres H Manidis M. Emergency communication: the discursive challenges facing emergency clinicians and patients in hospital emergency departments. Discourse Commun. 2008;2:271–98.

[29] Matthiessen CMIM. Applying systemic functional linguistics in healthcare contexts. Text Talk. 2013;33:437–67.

[30] Kuang Z Li SJ. A systematic functional perspective study on the discursive structure of the psychiatric doctor-patient Association. J Univ Sci Technol Beijing (Soc Sci Ed). 2019;1:8–15.

[31] Halliday MAK. An Introduction to Functional Grammar. London, UK: Edward Arnold; 1994.

[32] Halliday MAK Matthiessen CMIM. An Introduction to Functional Grammar. London, UK: Edward Arnold; 2004.

[33] Thompson G. Introducing Functional Grammar. London, UK: Hodder Education; 2004.

[34] Ware JE Snyder MK Wright WR. Development and validation of scales to measure patient satisfaction with health care services: volume 1 of a final report part B: results regarding scales constructed from the patient satisfaction questionnaire and measures of other health care perceptions. (NTIS No. PB 288-330). National Technical Information Service: Springfield, VA, USA; 1976.

[35] Baker R Whitfield M. Measuring patient satisfaction: a test of construct validity. Quality Health Care. 1992;12:104–9. 10.1136/qshc.1.2.104 PMC1054973 10172105

[36] Wilkin D Hallem L Duggett M. Measures of Need and Outcome for Primary Health Care. New York, USA: Oxford University Press; 1992.

[37] Carey RG Seibert JH. patient survey system to measure quality improvement: questionnaire reliability and validity. Med Care. 1993;31:834–45. 8366685 10.1097/00005650-199309000-00008

[38] Eggins S Slade D. Analyzing Casual Conversation. London, UK: Equinox; 2004.

[39] Hasan R. A semantic network for the analysis of messages in everyday talk between mothers and their children. Unpublished Ph. D. dissertation at Macquarie University, Sydney, Australia; 1983.

[40] Pérez SE González LL Acevedo IA. Attitudes and beliefs towards low back pain (LBP) among physiotherapists in Spain. Bull Faculty Phys Ther. 2022;27:1–7.

[41] Huang GW. Theory and Practice of Discourse Analysis. Shanghai, China: Shanghai Foreign Language Education Press; 2001.

[42] Ye N. Police interrogative discourse: A Study based on the holistic view of language classes. [dissertation thesis]. Hangzhou, China: Zhejiang University; 2010.

[43] Hall JA Dornan MC. What patients like about their medical care and how often they are asked: a meta-analysis of the satisfaction literature. Soc Sci Med. 1988;27:935–9. 3067368 10.1016/0277-9536(88)90284-5

[44] Jackson JL Chamberlin J Kroenke K. Predictors of patient satisfaction. Soc Sci Med. 2001;52:609–20. 11206657 10.1016/s0277-9536(00)00164-7

[45] Krupat E Fancey M Cleary PD. Information and its impact on satisfaction among surgical patients. Soc Sci Med. 2000;51:1817–25. 11128269 10.1016/s0277-9536(00)00113-1

[46] Hall JA Roter DL Katz NR. Meta-analysis of correlates of provider behavior in medical encounters. Med Care. 1988;26:657–75. 3292851 10.1097/00005650-198807000-00002

[47] Mishler EG. The Discourse of Medicine: Dialectics of Medical Interviews. NJ, USA: Ablex. Norwood; 1984.

[48] Liang HY. Doctor’s multiple identity construction in doctor-patient conversation. Foreign Stud. 2014;3:24–31.

[49] Sánchez Romero EA Lim T Villafañe JH. The influence of verbal suggestion on post-needling soreness and pain processing after dry needling treatment: an experimental study. Int J Environ Res Public Health. 2021;18:4206–. 33921101 10.3390/ijerph18084206 PMC8071378

[50] Barbas-Monjo MA Sánchez-Romero EA Villafañe JH Martínez-Rolando L Velasco García Cuevas J Cuenca-Zaldivar JN. Presence of differences in the radiofrequency parameters applied to complex pressure ulcers: a secondary analysis. Medicina (Kaunas). 2023;59:516–12. 36984517 10.3390/medicina59030516 PMC10059019

